# Multimodal imaging features to diagnose abdominal complications of sickle cell disease

**DOI:** 10.1007/s00261-026-05396-2

**Published:** 2026-02-10

**Authors:** Luana Lemos Alves, André Barboza Ferreira, Pedro Bernardo Berriel, Pietra Desirée Bourdon Fuentes Azevedo Vianna, Eleonora Salles-Silva, Antonio Luis Eiras-Araujo, Miriam Menna Barreto, Rosana Souza Rodrigues, Daniella Braz Parente

**Affiliations:** 1 Federal University of Rio de Janeiro, Rio de Janeiro, Brazil; 2 Fleury S.A. (Brazil), Rio de Janeiro, Brazil; 3 Rio de Janeiro State University, Rio de Janeiro, Brazil; 4 D’Or Institute for Research and Education, Rio de Janeiro, Brazil

**Keywords:** Sickle cell disease, Abdominal imaging, Magnetic resonance imaging, Computed tomography, Ultrasound, Vaso-occlusive crisis, Extramedullary hematopoiesis

## Abstract

Sickle cell disease (SCD) is a hereditary hemoglobinopathy, marked by chronic hemolysis and recurrent vaso-occlusive crises, which frequently leads to a wide range of abdominal complications. Sickled erythrocytes have reduced deformability and increased adhesion to vessel walls, leading to chronic vascular occlusion, inflammation, and hemolysis. Comprehensive imaging plays a pivotal role in the early detection and assessment of these complications, thereby mitigating morbidity and severe outcomes. Ultrasound (US), computed tomography (CT), and magnetic resonance imaging (MRI) are the three modalities commonly used on SCD patients to evaluate hepatosplenic involvement, renal pathology, pancreatobiliary disorders, gastrointestinal ischemia, extramedullary hematopoiesis, and genital abnormalities. The US is a first-line imaging tool due to its accessibility and low cost. CT offers high-resolution, cross-sectional imaging and is often utilized in emergency settings. MRI, with its superior soft tissue contrast, is excellent for problem-solving. A thorough understanding of the abdominal manifestations of SCD is essential for radiologists to provide accurate diagnoses, guide clinical decision-making, and reduce morbidity and mortality. This review highlights the most significant imaging findings from SCD abdominal complications, emphasizing their clinical implications, underlying pathophysiology, and radiological features in all three modalities.

## Introduction

Sickle cell disease (SCD) is a hereditary hemoglobinopathy caused by a mutation in the β-globin gene. The mutation causes the substitution of glutamic acid with valine at position 6 of the β-globin chain, leading to the formation of hemoglobin S (HbS). Under hypoxic conditions, HbS undergoes polymerization, altering the shape of erythrocytes and giving them a rigid, sickle-like appearance [1–3]. These sickled red blood cells (RBCs) have reduced deformability, increased adhesion to the vascular endothelium, and a shortened lifespan, contributing to a chronic state of vascular occlusion, inflammation, and intravascular hemolysis [4]. This process can result in ischemia, organ infarction and tissue necrosis, giving rise to a wide range of abdominal complications [5].

Radiologic imaging plays a critical role in the early detection and evaluation of these abdominal manifestations of SCD, timely and effective patient management, and reduced morbidity [5–9]. This review explores the radiologic findings associated with different abdominal complications of SCD, alongside a discussion of their underlying pathophysiologies and clinical implications.

## Discussion

### Pathophysiology

In SCD patients, environmental stressors such as hypoxia, acidosis, or dehydration trigger HbS polymerization and distort RBCs into stiff, sickle-like shapes. These cells adhere abnormally to the vascular endothelium and undergo premature destruction in the bloodstream (hemolysis). This process contributes to systemic inflammation and recurrent vascular obstruction [10]. The vaso-occlusive process causes ischemia and tissue necrosis, ultimately leading to progressive dysfunction of multiple abdominal organs. Accelerated hemolysis also causes chronic anemia and secondary iron overload, symptoms which are particularly evident in patients undergoing frequent blood transfusions [3].

**Fig. 1:**
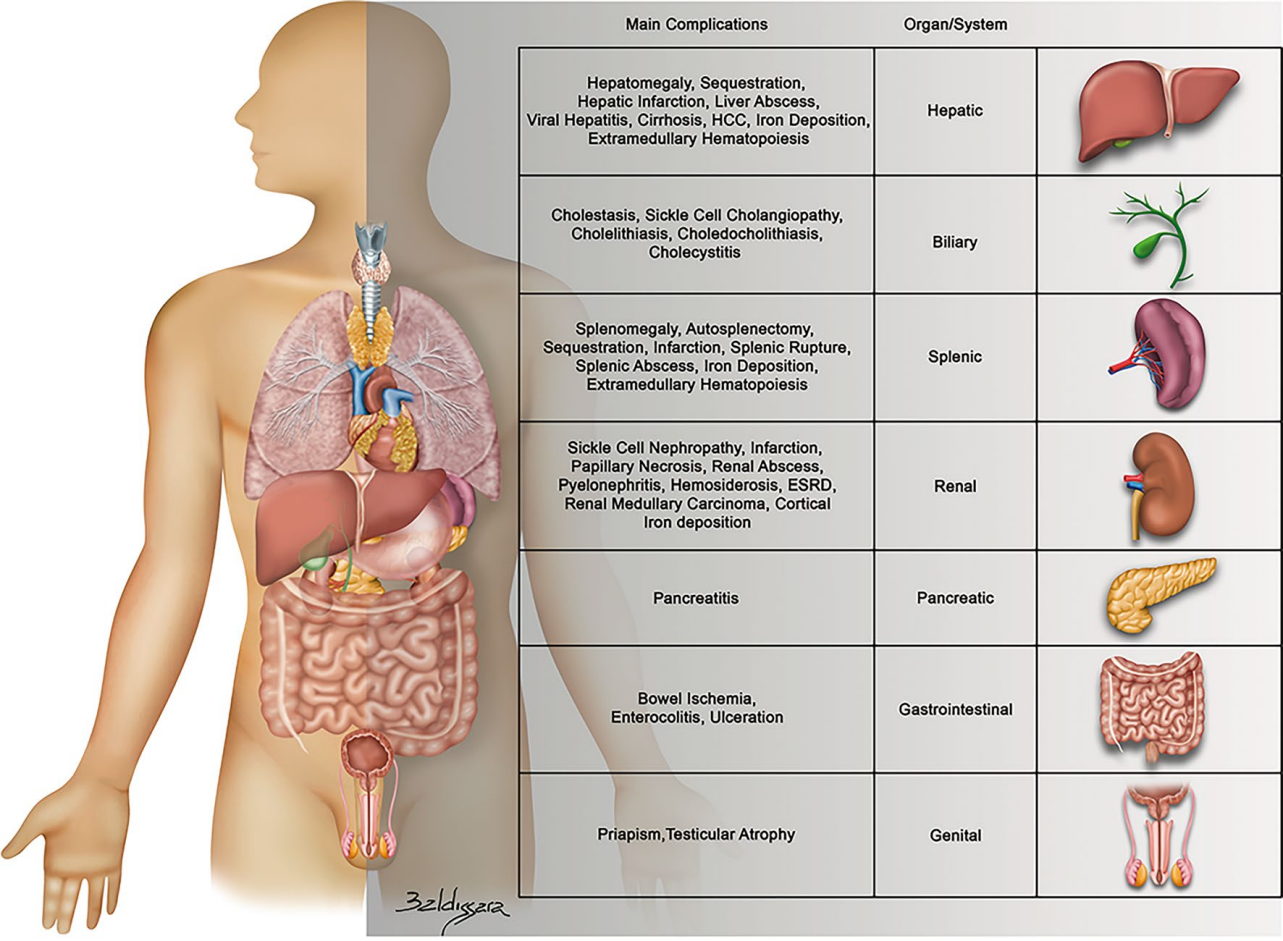
Overview of key abdominal manifestations in sickle cell disease (SCD). Illustration summarizing the main abdominal complications associated with SCD. These manifestations reflect the underlying pathophysiological mechanisms of vaso-occlusion, hemolysis, and iron overload characteristic of the disease

In abdominal organs, these pathological processes often result directly from vaso-occlusive mechanisms, including vascular congestion (engorgement of vessels due to impaired blood outflow) and vascular occlusion (complete obstruction of vascular flow), and inflammation, increasing the risk of complications such as splenic sequestration, infarction, necrosis, and hepatic or renal dysfunction [1, 6, 11]. Extramedullary hematopoiesis may also occur as a compensatory mechanism in response to ongoing erythrocyte destruction [12]. Figure 1 provides a visual overview and summarizes the main abdominal manifestations associated with SCD.

## Radiological manifestations

Ultrasonography (US), computed tomography (CT), and magnetic resonance imaging (MRI) play essential roles in evaluating these complications. US is widely accessible and particularly effective in detecting splenomegaly, cholelithiasis, and hepatic or renal involvement. CT provides high-resolution anatomical detail and is frequently used in emergency settings. MRI serves as a problem-solving tool and is especially valuable for diagnosing and quantifying iron deposition. The complementary use of these imaging modalities enhances diagnostic accuracy and supports optimal clinical management of the disease [5]. Table 1 summarizes the imaging approach to the major abdominal complications of sickle cell disease, highlighting first-line, gold standard, and emergency imaging modalities.

**Table 1:** Imaging modalities for major abdominal complications of sickle cell disease according to organ involvement and clinical setting

Organs	Complication	First-line	Gold standard	Emergency
Hepatic	Sequestration	CT	CT	CT
	Hepatic Infarction	CT	CT/MRI	CT
	Liver Abscess	CT	MRI	CT
Biliary	Cholelithiasis	US	US	US
	Choledocholithiasis	US	MRCP	US
	Cholecystitis	US	US	US
Splenic	Sequestration	CT	CT	CT
	Infarction	CT	CT/MRI	CT
	Splenic Rupture	CT	CT	CT
	Splenic Abscess	CT	MRI	CT
Renal	Infarction	CT	CT/MRI	CT
	Papillary Necrosis	CT	CT/MRI	CT
	Renal Abscess	CT	MRI	CT
Pancreatic	Pancreatitis	CT	CT	CT
Gastrointestinal	Bowel Ischemia	CT	CT	CT
	Enterocolitis	CT	CT	CT

Summary of preferred first-line, gold-standard, and emergency imaging modalities. In the emergency setting, CT is generally the primary modality, except for biliary complications where ultrasound plays a key role. CT remains the gold standard for most complications, while MRI, particularly diffusion-weighted imaging, can clarify challenging cases and improve characterization of abscesses, papillary necrosis, and renal infarction.

### Extramedullary hematopoiesis

In response to the accelerated destruction of RBCs and chronic anemia, the bone marrow undergoes hyperplasia as a compensatory mechanism, resulting in an expansion and increased activity of the medullary cavity. When this response is insufficient to meet the body’s hematopoietic demands, a secondary mechanism is triggered: the extramedullary proliferation of hematopoietic tissue [13].

**Fig. 2:**
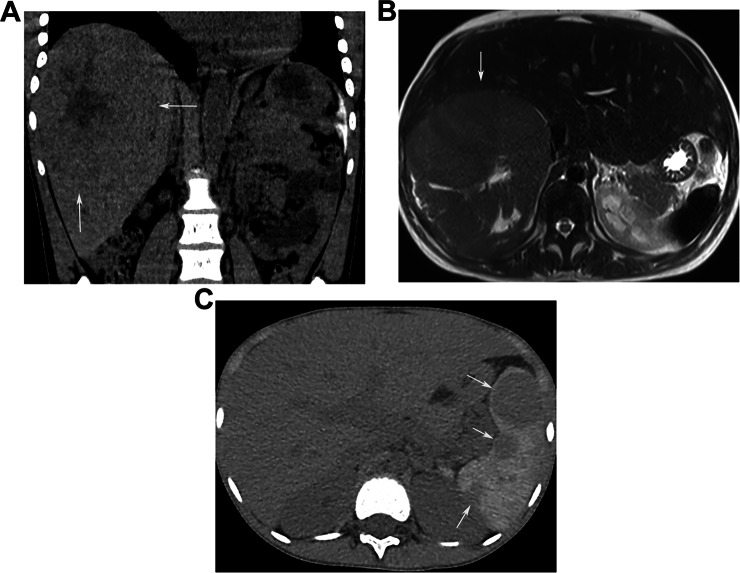
**a–c** Extramedullary hematopoiesis involving different abdominal organs. **a** Non-contrast CT image of a 28-year-old male with a history of recurrent hospitalizations due to vaso-occlusive pain episodes refractory to opioid therapy. The scan reveals a large hepatic mass with a central hypodense region (arrow), subsequently confirmed as extramedullary hematopoiesis on histopathological analysis. **b** Axial T2-weighted MRI of a 34-year-old male with a history of cholecystectomy, childhood ischemic stroke, recurrent vaso-occlusive pain crises, and chronic osteomyelitis. The image demonstrates a large right adrenal mass (arrow), with histopathological analysis confirming extramedullary hematopoiesis. **c** Non-contrast CT scan of a 45-year-old male with long-standing sickle cell disease and multiple associated complications. The image reveals multiple splenic nodules (arrows), consistent with extramedullary hematopoiesis

Extramedullary hematopoiesis (EMH) may present as soft tissue masses in various regions of the body, most commonly in the paravertebral area and thorax. In the abdomen, its most frequent manifestation is hepatosplenomegaly. Hepatic and splenic nodules are also possible. Rare EMH sites include the perirenal space, omentum, mesentery, peritoneum, and skin [13, 14]. Figure 2A–C illustrate cases of EMH in the liver, the right adrenal gland, and the spleen respectively.

On US, these lesions often appear hypoechoic when located within solid organs, and may be associated with hepatomegaly or splenomegaly. On CT, they typically present as well-defined regions with mild contrast enhancement. MRI generally reveals masses that are isointense on T1-weighted images, are isointense or slightly hyperintense on T2-weighted images, and exhibit subtle enhancement post-contrast. Fat and iron content may also be present [13, 15, 16]. The differential diagnosis for this imagery includes other conditions such as lymphoma, leukemia, metastases, and rarely sarcoma. A combination of clinical history, imaging studies, and histopathological examination is essential to distinguish EMH. MRI with diffusion-weighted imaging can help, in that neoplastic masses typically demonstrate restricted diffusion while EMH lesions usually do not [13, 17].

### The spleen

**Fig. 3:**
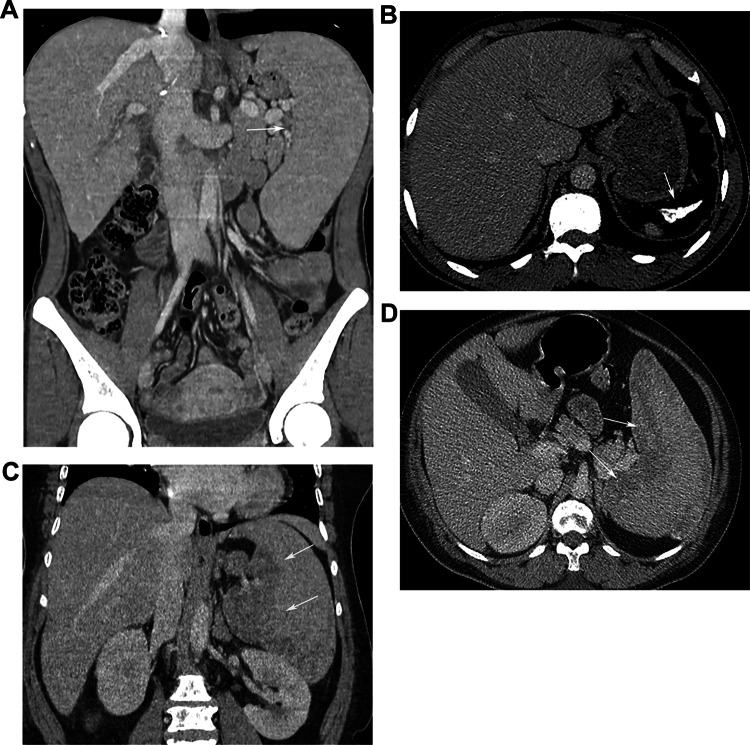

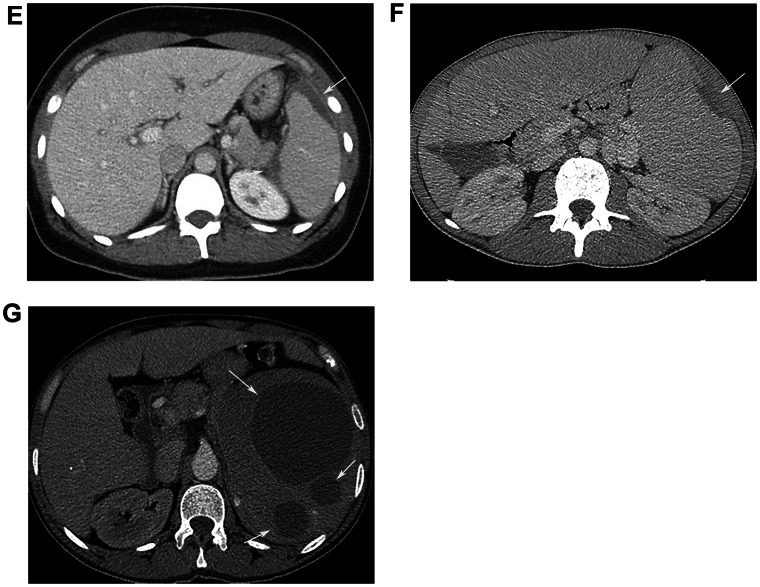
**a–g** Spectrum of splenic manifestations in sickle cell disease. **a** Coronal reformation of a post-contrast CT scan in a 26-year-old female with a history of liver transplantation due to autoimmune hepatitis-induced cirrhosis, presenting with acute abdominal pain. The image demonstrates splenomegaly (arrow). **b** Post-contrast CT scan of a 30-year-old male with sickle cell disease. The image demonstrates a small, calcified spleen (arrow), consistent with autosplenectomy. **c**, **d** Post-contrast CT scan of a 58-year-old female with sickle cell disease presenting with acute abdominal pain. The image demonstrates splenomegaly and hypovascular areas within the spleen (arrows), consistent with splenic sequestration. **e** Post-contrast CT scan in a patient with sickle cell disease demonstrates a crescent-shaped subcapsular perisplenic fluid collection (arrows), consistent with a perisplenic hematoma secondary to splenic rupture. **f** Post-contrast CT scan of a 30-year-old male presenting with a pain crisis, reporting severe pain in the arms, chest, and right leg, as well as a history of chronic cough. The image demonstrates an enlarged spleen with a peripheral wedge-shaped hypovascular lesion and mild capsular retraction (arrow), findings consistent with splenic infarction. **g** Post-contrast CT scan of a 44-year-old male presenting with severe pain in the right upper limb and left hemithorax, accompanied by fever and leukocytosis. The image demonstrates hypodense, fluid-attenuation lesions within the spleen (arrows), suggestive of splenic abscesses

The spleen is one of the most commonly affected organs in SCD, exhibiting a broad spectrum of radiological manifestations [5–7]. Splenic changes result directly from the vascular congestion and vaso-occlusive mechanisms. Furthermore, the spleen plays a crucial role in filtering abnormal RBCs. In SCD, splenomegaly is observed in approximately 10% of patients over the age of 10. Sickled RBCs can become trapped in the spleen, leading to congestion and enlargement (Fig. 3A) [18].

Autosplenectomy in SCD occurs due to recurrent episodes of vascular occlusion and infarction. Over time, the spleen undergoes progressive functional loss due to fibrogenesis within the red pulp, which develops in response to repeated entrapment and hemorrhage of RBCs [19]. Imaging features such as a small, calcified spleen with low signal intensity indicate fibrotic and hemorrhagic remodeling within the organ. This hyposplenism results in increased vulnerability to infections by encapsulated bacteria, although islands of functional splenic tissue may persist (Fig. 3B) [20].

Splenic sequestration occurs more frequently in children with hemoglobin SS (HbSS) and is characterized by the sudden pooling of blood in the spleen, resulting in acute splenomegaly and severe anemia (Fig. 3C and D). This event is often preceded by sickle cell crisis, infection, or acute chest syndrome, and may progress to cardiovascular collapse, splenic rupture, or death. Imaging can reveal rapid splenomegaly with signs of intraparenchymal or perisplenic hemorrhage (Fig. 3E) [7].

Splenic infarctions result from chronic vascular occlusion and contribute to the progressive atrophy of the spleen, with replacement of the parenchyma by fibrous tissue, calcium, and hemosiderin deposits. On contrast-enhanced CT, infarcts typically appear as peripheral, wedge-shaped hypovascular areas (Fig. 3F). On US, they present as heterogeneous regions with hypoechoic foci. MRI may show hyperintense areas on T1-weighted images indicating intraparenchymal hemorrhage. The presence of perisplenic fluid or complex peritoneal collections should raise suspicion for splenic rupture [21].

Splenic abscesses can arise from superinfection of infarcted areas, leading to fever and left upper quadrant pain. On US, they appear as complex hypoechoic lesions, which may be unilocular or multiseptated, sometimes containing gas, seen as echogenic foci with posterior acoustic shadowing. On contrast-enhanced CT and MRI, splenic abscesses appear as low-attenuation, multiseptated collections that are hyperintense on T2-weighted images. They are typically surrounded by edema, peripheral enhancement, and exhibit marked diffusion restriction (Fig. 3G) [6].

Differentiating a splenic abscess from a splenic infarct can be challenging, particularly in advanced stages when chronic changes alter normal imaging patterns. Discriminatory imaging features include the presence of peripheral enhancement and restricted diffusion in abscesses, or sharper, avascular margins without internal septations in infarcts. These imaging features must be combined with clinical presentation, inflammation markers and serial imaging to prevent misdiagnosis [22, 23].

### The kidneys

The kidneys are commonly affected in SCD due to hypoxia and the acidic environment of the renal medulla, both of which promote the sickling of RBCs. Sickle RBCs in the kidney can result in glomerular and tubular injury [1]. Patients with SCD are also at increased risk for urinary tract infections and renal abscesses, due to tubular dysfunction and alkaline urine, both of which promote bacterial growth [24].

Sickle cell nephropathy may lead to progressive renal ischemia, cortical infarctions, and papillary necrosis, ultimately impairing renal function and increasing the risk of chronic kidney disease [2, 3]. US findings may include increased echogenicity of the renal cortex compared to the adjacent liver or spleen, loss of corticomedullary differentiation, and an elevated resistance index (> 0.75), typically indicating chronic parenchymal injury secondary to recurrent vaso-occlusive events [25]. Cortical hyperechogenicity also appears in conditions such as diabetic nephropathy, hypertensive nephropathy, and chronic pyelonephritis [26]. The clinical context and laboratory findings are essential for establishing the correct diagnosis [27].

In non-enhanced CT, chronic nephropathy can appear as cortical scarring and thinning of the renal parenchyma. Renal infarcts appear as subtle, wedge-shaped regions of hypoattenuation. Perirenal hematomas present as perirenal fluid collections [28]. Post-contrast CT images may show a delayed nephrogram; however, the use of intravenous contrast should be avoided in patients with renal insufficiency. Recent consensus statements and clinical guidelines recommend avoiding intravenous contrast in SCD patients with renal insufficiency due to the increased risk of contrast-associated acute kidney injury and the potential for exacerbating sickling events in the setting of dehydration and renal dysfunction. The American College of Radiology and the National Kidney Foundation jointly recommend prophylactic intravenous normal saline hydration for patients with eGFR < 30 mL/min/1.73 m² or acute kidney injury who are not on dialysis and consider hydration for those with eGFR 30–44 mL/min/1.73 m² in high-risk circumstances [29–31].

Earlier reports described increased erythrocyte sickling with older hyperosmolar ionic iodinated contrast agents. Current evidence indicates that modern low- and iso-osmolar nonionic contrast media are associated with a low incidence of clinically significant adverse events in patients with SCD [32].

**Fig. 4:**
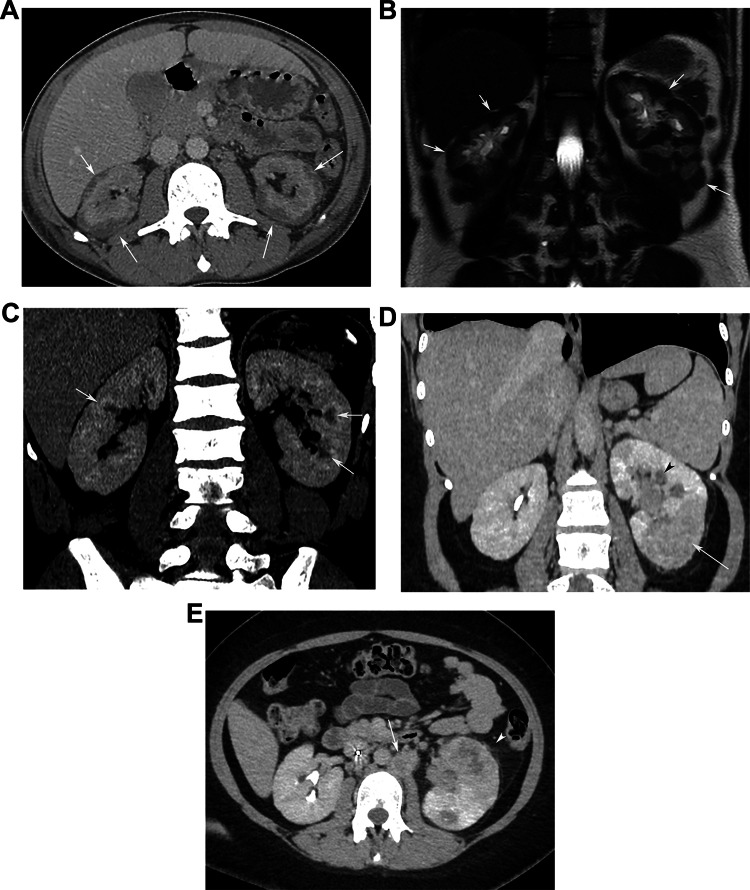
**a**–**e** Renal manifestations of sickle cell disease. **a** Post-contrast CT scan of a 34-year-old male with sickle cell disease, presenting with epigastric abdominal pain and elevated serum amylase and lipase levels, initially managed as severe pancreatitis. The clinical course was complicated by worsening hyperbilirubinemia and acute kidney injury requiring dialysis. The scan demonstrates bilateral renal cortical necrosis (arrows). **b** Coronal T2-weighted MRI of a 42-year-old male with long-standing sickle cell disease and multiple complications. The image demonstrates diffusely hypointense signal intensity in the renal cortex bilaterally (arrows), consistent with renal hemosiderosis. The liver and spleen also show diffuse low signal intensity on T2-weighted imaging, indicative of iron deposition. **c** Equilibrium-phase post-contrast CT scan of a 30-year-old male presenting with intense pain involving both upper and lower extremities, as well as the lumbar region. Past medical history includes avascular necrosis of the femoral head. The image shows bilateral triangular-shaped areas with reduced contrast enhancement in the renal medulla, consistent with papillary necrosis. **d**, **e** Equilibrium-phase post-contrast CT scan of a 26-year-old female with sickle cell trait, presenting to the emergency department with abdominal pain. The scan demonstrates an infiltrative hypovascular renal mass (arrow in **d**) associated with collecting system dilatation (arrowhead in **d**), perinephric infiltration (arrowhead in **e**), and lymphadenopathy (arrow in **e**)

Figure 4A shows a cortical renal infarction on contrast-enhanced CT. Differentiating renal infarction from pyelonephritis can be challenging. A sharply demarcated wedge-shaped area with its base oriented toward the renal capsule favors renal infarction. In contrast, pyelonephritis may present with blurred lesion borders and preservation of peripheral cortical rim enhancement. In some cases, intraparenchymal hemorrhage may lead to the formation of a perirenal hematoma [7]. MRI may further demonstrate loss of corticomedullary differentiation and cortical iron deposition (Fig. 4B) [5].

Papillary necrosis arises from vaso-occlusion and chronic ischemia. It may present clinically with painless hematuria, anemia, hydronephrosis or oliguria [33]. On contrast-enhanced imaging, hypoattenuation at the tips of the renal pyramids may be observed. The characteristic signs are sometimes described as “ball-on-tee” or “lobster claw”, highlighting the contour of the necrotic papillae (Fig. 4C) [5].

Renal medullary carcinoma is a rare but aggressive malignancy that occurs almost exclusively in individuals with the sickle cell trait. It presents as an infiltrative mass arising from the renal medulla and may cause flank pain, hematuria, and weight loss. On US, it appears as a heterogeneous mass; on CT and MRI, it typically appears as a hypovascular lesion associated with collecting system dilatation, perinephric infiltration, and lymphadenopathy. The prognosis is poor, and treatment often involves surgery, chemotherapy and immunotherapy (Fig. 4D and E) [5].

### The liver

The liver is frequently impacted by SCD complications related to chronic hemolysis, vaso-occlusive crises, and iron overload from repeated blood transfusions. Manifestations include hepatomegaly, acute hepatic sequestration, hepatic infarctions, iron deposition, viral hepatitis, and cirrhosis. In advanced stages, these complications may rarely progress to hepatocellular carcinoma [34–37].

**Fig. 5:**
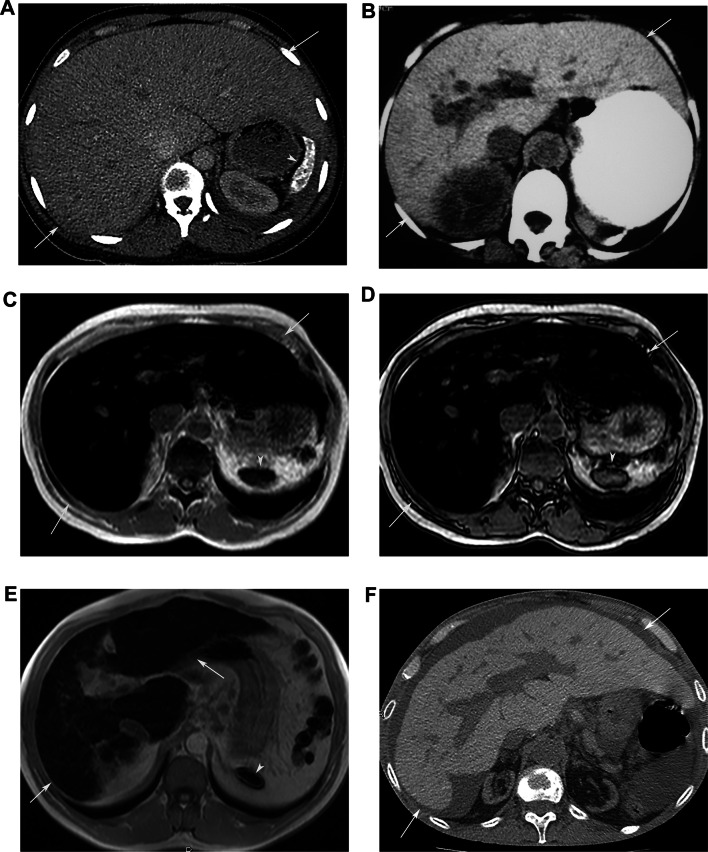
**a–f** Hepatic manifestations of sickle cell disease. **a** Post-contrast CT scan of a 26-year-old patient with sickle cell disease presenting with a diffuse pain crisis predominantly affecting the limbs, two recent episodes of fever, and choluria associated with worsening jaundice. The scan demonstrates hepatomegaly due to hepatic congestion (arrows). Evidence of autosplenectomy is also noted (arrowhead). **b** Non-contrast CT scan of a 41-year-old patient with long-standing sickle cell disease demonstrating diffusely increased hepatic attenuation (arrows), consistent with secondary hemochromatosis. **c**, **d** Axial T1-weighted in-phase (**c**) and out-of-phase (**d**) MRI images of a 52-year-old patient with long-standing sickle cell disease. The images demonstrate diffuse hypointense signal in the liver (arrow) and spleen (arrowhead), with a marked signal drop on in-phase imaging, findings suggestive of iron overload. **e** T1-weighted MRI of a 51-year-old male with sickle cell disease, presenting with jaundice, disorientation, and somnolence. The image demonstrates features of chronic liver disease, including relative hypertrophy of segments II, III, and the caudate lobe; hypotrophy of segment IV; irregular hepatic contours; and widened fissures (arrows). Diffuse low signal intensity of the liver reflects iron deposition. Evidence of autosplenectomy is also seen (arrowhead). **f** Non-contrast CT scan of a 62-year-old male with sickle cell disease, presenting with dyspnea on minimal exertion and orthopnea. Past medical history includes priapism, sickle cell nephropathy, and osteonecrosis of the femoral head. The image demonstrates features of chronic liver disease, including irregular hepatic contours and hypertrophy of the left and caudate lobes (arrows). Ascites is also present

Hepatomegaly is one of the earliest and most common findings. It may be associated with vascular congestion, iron overload, or infiltration by extramedullary hematopoietic tissue (Fig. 5A) [6]. On US, hepatomegaly presents as increased liver volume with a homogeneous or slightly heterogeneous echotexture [5].

Hepatic iron overload (hemosiderosis) is a consequence of chronic hemolysis and frequent blood transfusions. It may lead to liver dysfunction, cirrhosis, and an elevated risk of hepatocellular carcinoma. On US, it may manifest as increased hepatic echogenicity. On CT, the liver may show markedly increased attenuation, often exceeding 80 Hounsfield Units (HU) (Fig. 5B) [37, 38]. MRI of the liver typically reveals a diffuse signal drop of the gradient-echo in-phase compared to out-of-phase images, and low signal intensity of T2-weighted images due to the longer echo time [5, 6].

The American Society of Hematology recommends regular assessment of liver iron concentration (LIC) by MRI in chronically transfused patients with sickle cell disease because it provides a more accurate assessment of iron overload than serum ferritin, which can be confounded by inflammation and other factors in sickle cell disease [39]. The Society of Abdominal Radiology (SAR) and the European Society of Gastrointestinal and Abdominal Radiology (ESGAR) jointly recommend noninvasive MRI-based quantification of LIC as the preferred method, emphasizing that LIC is linearly related to total body iron stores and is the best surrogate for assessing iron overload in transfusion-dependent hemoglobinopathies, including sickle cell disease. MRI-R2 and MRI-T2* techniques are validated against liver biopsy, with MRI-T2* offering the advantage of rapid acquisition and assessment of extrahepatic iron deposition. Thresholds for intervention are well established: LIC < 1.8 mg/g is normal, 3.2–7 mg/g is mild overload, 7–15 mg/g is moderate, and > 15 mg/g is severe, with increased risk of hepatic and systemic complications. The multi-echo gradient echo sequence produces T2*, R2* and fat fraction maps, allowing calculation of LIC and the percentage of steatosis. This technique is routinely used in clinical practice [40]. Iron overload in the liver and spleen is illustrated in Fig. 5C and E.

Additionally, US elastography and MR elastography can assess liver stiffness and are the methods of choice for diagnosing and quantifying liver fibrosis. These methods can be used to determine a prognosis, monitor progress, assess a treatment response, and detect portal hypertension. While US elastography is simple, rapid, and more widely available, MR elastography is able to evaluate a larger volume of liver parenchyma [41–43]. Noninvasive liver fibrosis estimation by elastography has been recently used to diagnose and stage fibrosis, reducing the need for invasive biopsy in patients with SCD [44, 45].

Acute hepatic sequestration is a life-threatening complication marked by massive sequestration of RBCs in the liver. This results in sudden hepatomegaly, intense right upper quadrant pain, and pallor, potentially progressing rapidly to severe anemia or hypovolemic shock due to reticuloendothelial uptake of erythrocytes [34, 36]. US may show a heterogeneous echotexture, which is sometimes associated with portal vein thrombosis. CT may reveal patchy hypoattenuation consistent with ischemia or segmental hypoperfusion, while MRI may show high T2 signal in affected areas and high T1 signal if recent hemorrhage is present [6].

Hepatic infarctions result from the occlusion of small intrahepatic vessels and appear as hypoattenuated, irregular or wedge-shaped lesions on contrast-enhanced CT, often surrounded by perilesional edema. On MRI, infarcts typically appear hypointense on T1 and hyperintense on T2, with associated diffusion restriction. Differentiating hepatic infarcts from hepatic abscesses can be challenging, and peripheral enhancement is more indicative of infection [5, 7].

Viral hepatitis, which may develop in chronically transfused patients, can progress to cirrhosis as a late-stage complication [46]. US may reveal heterogeneous hepatic echotexture and signs of portal hypertension, such as splenomegaly and venous collaterals. On CT and MRI, features of chronic liver disease include parenchymal fibrosis, caudate lobe hypertrophy, and medial segment atrophy of the left lobe (Fig. 5F) [5, 6].

Focal hepatic lesions, such as dysplastic nodules, hepatocellular carcinoma, perfusion defects, and benign entities can occur in SCD, especially in patients with cirrhosis secondary to hemosiderosis or chronic viral hepatitis [5]. Distinguishing benign from malignant hepatic lesions can be challenging in SCD, especially when there is iron overload. Regenerative and dysplastic nodules show enhancement similar to the surrounding parenchyma. Hepatocellular carcinoma (HCC) is rare in this population, but should be considered when there is a nodule with arterial phase hyperenhancement, washout, and an enhancing capsule on CT and MRI [5, 36]. Hepatobiliary contrast agents and diffusion-weighted imaging (DWI) are also useful in differentiating HCC, which appears hypointense in the hepatobiliary phase and may demonstrate restricted diffusion [47].

### The gallbladder and bile ducts

**Fig. 6:**
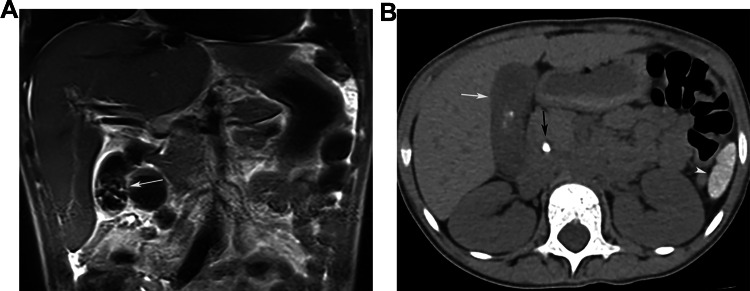
**a**,** b** Biliary manifestations of sickle cell disease. **a** Coronal T2-weighted MRI of a 36-year-old male presenting with jaundice, diarrhea, and vomiting, followed by progressive disorientation over two days. The image shows a gallbladder filled with gallstones (arrow). **b** Non-contrast CT scan of a 35-year-old female presenting with fever, acute abdominal pain, and nausea. The image demonstrates a thick-walled gallbladder containing calculi, consistent with cholecystitis (white arrow). A calculus is also visualized in the common bile duct, indicating choledocholithiasis (black arrow). Evidence of autosplenectomy is noted (arrowhead)

Cholelithiasis is a common complication of SCD, affecting up to 25% of patients [48]. It is directly related to chronic hemolysis, which increases the excretion of indirect bilirubin, promoting the precipitation of bilirubinate crystals [36]. Unlike gallstones in the general population, which are predominantly cholesterol-based, those in SCD are primarily composed of calcium bilirubinate and typically pigmented. They are radiopaque in approximately 50% of cases [49]. On US, these stones appear as echogenic structures with posterior acoustic shadowing. CT is insensitive to cholelithiasis, visualizing only dense stones [6]. MRI is highly sensitive and can detect stones as low-signal foci on T2-weighted images (Fig. 6A) [5].

Cholecystitis, or inflammation of the gallbladder, is usually caused by stone impaction in the gallbladder infundibulum. US findings can include gallbladder wall thickening (> 3 mm), pericholecystic fluid, and a positive sonographic Murphy’s sign [9].

Choledocholithiasis, which occurs in up to 18% of patients with cholelithiasis (Fig. 6B), can lead to biliary obstruction and ascending cholangitis. Magnetic resonance cholangiopancreatography (MRCP) is the gold standard for diagnosis, as it can image both filling defects within the common bile duct and upstream biliary dilation. On US, choledocholithiasis may be suggested by dilation of the common bile duct, although direct visualization of stones is often limited by bowel gas. CT has lower sensitivity than MRCP for detecting bile duct stones. Cholangitis, a severe infectious complication, typically presents with fever, jaundice, and right upper quadrant pain. On US and CT, findings include biliary duct dilation and wall thickening, while MRI may show high T2 signal around the ducts, indicating periductal inflammation [5–7].

Sickle cell cholangiopathy is a form of biliary tract disease caused by recurrent vaso-occlusive crises leading to ischemia in the terminal branches of the biliary tree. Initially, this manifests as biliary dilation, but it may progress to multifocal strictures involving both intrahepatic and extrahepatic ducts. MRCP typically reveals segmental or diffuse dilation, ductal irregularities, and strictures. Differential diagnoses include sclerosing cholangitis and biliary neoplasms, so excluding choledocholithiasis is an important step. In advanced stages, sickle cell cholangiopathy can evolve into recurrent cholangitis and biliary cirrhosis, underlining the need for early imaging diagnosis [36, 50–52].

### The pancreas

The pancreas is rarely involved in sickle cell anemia. When affected, acute pancreatitis is the most frequent manifestation, usually secondary to choledocholithiasis or ischemic injury from microvascular occlusion [53].

On contrast-enhanced CT, uncomplicated pancreatitis typically presents as mild pancreatic enlargement with preserved parenchymal enhancement and peripancreatic inflammatory changes, which may include fluid collections [7].

On MRI, the pancreatic parenchyma may exhibit reduced T1 signal intensity compared to the normal pattern. T2-weighted images often show a hyperintense signal, along with reduced and heterogeneous enhancement of the parenchyma in the early post-contrast phases. Peripancreatic inflammatory infiltration and fluid collections may also be present. In complicated cases, both CT and MRI may demonstrate non-enhancing areas, indicating pancreatic or peripancreatic necrosis, fat necrosis in surrounding tissues, and signs of deep vein thrombosis [5].

Although most patients respond well to conservative treatment, early imaging detection of complications is essential, especially in pancreatitis related to biliary lithiasis. Elective cholecystectomy can be considered once the acute episode resolves [7].

### The gastrointestinal tract

Vaso-occlusive crises in sickle cell anemia can affect the gastrointestinal tract, potentially leading to mesenteric and enteric ischemia. The post-capillary venous microvasculature is the primary site of involvement, resulting in intestinal wall thickening and submucosal edema [54].

On contrast-enhanced CT, a characteristic finding is the “target sign”, defined by prominent enhancement of the inner intestinal wall with a hypodense submucosal layer due to edema [5]. Additional imaging features may include mesenteric fat stranding and mild ascites [8].

Although intestinal ischemia in SCD is usually microvascular, rare cases of mesenteric venous thrombosis have been reported [7]. The ischemic involvement may be segmental or diffuse, with a predilection for the colon, owing to its relatively lower blood flow [8].

Potential complications include intestinal necrosis, perforation, intra-abdominal abscess formation, and peritonitis. Other manifestations such as paralytic ileus and gastric ischemic ulcers may also occur. Early detection of these complications is critical to prevent life-threatening outcomes [8].

### The genitals

Priapism is a low-flow vaso-occlusive complication of SCD, affecting approximately 30% to 40% of male patients over their lifetime [55]. On Doppler US, findings may include thrombosis of the corpora cavernosa or corpus spongiosum with reduced blood flow. On CT and MRI, the penis may appear erect. MRI can also demonstrate intracavernous hemorrhage, characterized by high signal intensity on T1-weighted images within the cavernous bodies [6].

Testicular atrophy occurs in up to 24% of patients, often due to vaso-occlusion or the chronic use of hydroxyurea [56]. On US, CT, and MRI, the testes appear heterogeneous and reduced in size. Imaging is crucial for differential diagnosis and for monitoring potential endocrine repercussions [5].

### Novel techniques

Novel imaging techniques are promising for early diagnosis and organ-targeted treatment of SCD. Functional MRI methods, including diffusion tensor imaging (DTI), show potential in characterizing microstructural changes to the kidney, allowing earlier detection of subclinical kidney injury [57, 58]. Additionally, PET-CT (Positron Emission Tomography) is providing valuable insights into the metabolic and functional status of affected organs. However, its routine use in clinical practice requires further validation [59].

Photon-counting CT (PCCT) is an emerging technology that offers higher spatial resolution, improved tissue contrast, and reduced radiation exposure compared to conventional CT. By counting individual X-ray photons and analyzing their energy, PCCT may enhance visualization of parenchymal and subtle vascular abnormalities [60]. In SCD, this technology could improve the detection of small organ infarctions, early vascular disease, and iron-related tissue findings. However, further prospective studies are warranted to determine the diagnostic and prognostic value of this modality in clinical practice.

## Conclusion

Imaging plays a crucial role in the diagnosis, management, and follow-up of patients with SCD, who experience a wide range of abdominal complications. The use of US, CT, and MRI as complementary modalities enables early detection of potentially severe manifestations, guides clinical decision-making, and contributes to effective treatment strategies. Timely radiologic evaluation is essential for reducing both morbidity and mortality, ultimately improving patient outcomes.

## Data Availability

No datasets were generated or analysed during the current study.
